# Comparing the efficacy of concomitant treatment of resistance exercise and creatine monohydrate versus multiple individual therapies in age related sarcopenia

**DOI:** 10.1038/s41598-024-59884-w

**Published:** 2024-04-29

**Authors:** Eman I. Elgizawy, Ghada S. Amer, Eman A. Ali, Fatma S. Alqalashy, Marwa M. Ibrahim, Asmaa A. Abdel Latif, Anwar M. Shaban

**Affiliations:** 1 Medical Physiology Department, Faculty of Medicine, Menoufia University, Yassin Abd El Ghafar St., Shebin El Kom, Menoufia 32511 Egypt; 2 Clinical Pharmacology Department, Faculty of Medicine, Menoufia University, Shebin El Kom, Menoufia Egypt; 3 Pathology Department, Faculty of Medicine, Menoufia University, Shebin El Kom, Menoufia Egypt; 4 Medical Biochemistry and Molecular Biology Department, Faculty of Medicine, Menoufia University, Shebin El Kom, Menoufia Egypt; 5 Public Health and Community Medicine Department, Faculty of Medicine, Menoufia University, Shebin El Kom, Menoufia Egypt

**Keywords:** Aged rats, Exercise, Coenzyme Q 10, Creatine monohydrate, Oxidative stress, Biochemistry, Cell biology, Physiology

## Abstract

Aging-related sarcopenia is a degenerative loss of strength and skeletal muscle mass that impairs quality of life. Evaluating NUDT3 gene and myogenin expression as new diagnostic tools in sarcopenia. Also, comparing the concomitant treatment of resistance exercise (EX) and creatine monohydrate (CrM) versus single therapy by EX, coenzyme Q10 (CoQ10), and CrM using aged rats. Sixty male rats were equally divided into groups. The control group, aging group, EX-treated group, the CoQ10 group were administered (500 mg/kg) of CoQ10, the CrM group supplied (0.3 mg/kg of CrM), and a group of CrM concomitant with resistance exercise. Serum lipid profiles, certain antioxidant markers, electromyography (EMG), nudix hydrolase 3 (NUDT3) expression, creatine kinase (CK), and sarcopenic index markers were measured after 12 weeks. The gastrocnemius muscle was stained with hematoxylin–eosin (H&E) and myogenin. The EX-CrM combination showed significant improvement in serum lipid profile, antioxidant markers, EMG, NUDT3 gene, myogenin expression, CK, and sarcopenic index markers from other groups. The NUDT3 gene and myogenin expression have proven efficient as diagnostic tools for sarcopenia. Concomitant treatment of CrM and EX is preferable to individual therapy because it reduces inflammation, improves the lipid serum profile, promotes muscle regeneration, and thus has the potential to improve sarcopenia.

## Introduction

Sarcopenia is a physiological age-related condition recognized by attenuation of the physical functions of the muscle and loss of its bulk (strength of performance) without a corresponding decrease in body weight^1,2^. Additionally, it has adverse outcomes, including falls, fractures, and physical disability. This definition has been established by the European Working Group on Sarcopenia in Older People (EWGSOP)^3^.

Starting at age fifty, sarcopenia prevalence in the population ranges from 2 to 9%, and with recent changes in aging-related lifestyles around the world, the prevalence rate is rising quickly^4^. It is a significant disorder in geriatric medicine as it leads to metabolic disturbance and increases the risk of many disabilities that impair life quality^5^.

Ageing is caused by DNA oxidation (many genetic loci) and cumulative oxidative stress, as well as low-grade inflammation^6,7^*,* generating a disturbed skeletal muscle homeostasis that eventually leads to increased reactive oxygen species (ROS), defective autophagy, and myocyte apoptosis^8^. So, sarcopenia is considered an ordinary sequel of aging^2^.

Muscle mass, muscle strength, and physical performance are the three axes that sarcopenia diagnosis is based on by EWGSOP in 2010^9^. The diagnosis was confirmed by the presence of low muscle quality and quantity. However, the use of muscle quality, which is described by the micro- and macroscopic aspects of muscle composition, as a primary parameter remains challenging due to technological limitations^10^.

This research tries new lines in diagnosis and evaluation as NUDT3. It is one of the genes identified to be enriched in skeletal muscles by genotype tissue expression (GTEx) tissue analysis for lean body mass (LBM). which is studied concerning oxidative stress, inflammation, and sarcopenia^11^. Nudix protein families serve as homeostatic checkpoints at critical phases in inositol phosphate metabolic pathways, including NUDT3^12^. Also, myogenesis is skeletal muscle cell differentiation. MyoD, a myogenic regulatory factor, induces the transcription of cyclin-dependent kinase inhibitors to stop proliferating myoblasts' cell cycle^13^ and early skeletal muscle-specific genes, including myogenin^14^. MyoD and myogenin trigger later skeletal muscle-specific genes that assist myoblasts in entering the myogenic differentiation pathway^15^.

Management strategies for sarcopenia have evolved over time, encompassing both non-pharmacological and pharmacological approaches. These strategies have focused on exercise interventions and nutritional aspects, which may involve supplementation^16^.

Exercise (EX) is known for boosting cellular, tissue, and organ metabolism^17,18^. Resistance training builds muscle and improves function, while aerobic exercise resists cardiovascular disease better^19,20^.

When performing resistance exercises, skeletal muscle produces creatine monohydrate (CrM), which serves as a vital source of energy, adenosine diphosphate (ADP), and an acid buffer^21^. Additionally, it offers the phosphate required for the resynthesis of adenosine triphosphate (ATP), allowing its rapid turnover and sustained maximum contractions. However, recent research^17^ has shown that using CrM supplements and endurance training can raise citrate synthase levels^22,23^.

The only endogenous fat-soluble antioxidant generated de novo in the body is ubiquinone, or CoQ10. Prominent functions include mitochondrial bioenergetics and cell signaling gene synthesis^24^. The reduced form of CoQ10, a powerful antioxidant, may recycle mitochondrial tocopherols into alpha-tocopherols, replenishing vitamin E^25^. Additionally, CoQ10 may reduce oxidative stress^26^.

This study aimed to investigate the NUDT3 gene which has not been studied before, myogenin expression as evaluation tools and to identify new treatment approaches for delaying age-related sarcopenia. The study compared the results of EX, CrM, and CoQ10 individually as well as the combined effects of exercise and CrM on aging rats.

## Materials and methods

All experimental protocols and methodology were carried out following the Guide for the Care and Use of Laboratory Animals^27^. The local ethical committee (Menofia University's Animal Care and Use Committee) authorized the experimental protocol with approval code 5/2023PHYS16. This study followed Animal Research: Reporting of In Vivo Experiments (ARRIVE) guidelines. The study was conducted between November 2022 and March 2023.

### Animals

#### Sample size estimation

The sample size calculation for this experimental study rendered 60 rats (10 rats in each group) based on 70% and 30% as a percentage of exposed (sarcopenia) and unexposed outcomes, respectively, according to a previous study^28^. A two-sided 95% significance level with 80% power and a 1:1 exposed-unexposed ratio is required. G*Power (3.1.9.2; Germany) calculated this sample size.

This investigation utilized sixty adult male Wistar albino rats matched for weight and age between 250 and 300 g. During the investigation, each rat pair was housed in a single, well-ventilated cage with free access to food and water. Exposing them to average room temperature, humidity, and light/dark cycles. Before the commencement of the study, rats were conditioned for 2 weeks.

### Experimental design

Rats were randomly divided into six groups (10 per group):

*Group I*: Control group (CTR): Adult rats (3 months of age) received distilled water (DW) via oral gavage^29^.

*Group II*: Aged group: Aged rats (age 20–24 months) received DW via oral gavage^30^.

*Group III*: Exercise (EX)-treated group (EX): Aged rats (age 20–24 months) received DW via oral gavage. Each rat was subjected to ladder climbing resistance exercise for 15 min three times per week for 12 weeks^31^.

*Group IV*: CoQ_10_-treated group: Aged rats received CoQ_10_ capsules (CoQ-10 100 mg, Natrol Co., Chatsworth, CA, USA) dissolved in DW at a dose of 500 mg/kg/day with oral gavage (500 mg diluted in DW to obtain 5 ml/kg) for 12 weeks^32^.

‏*Group V*: CrM-treated group: Aged rats received CrM (8.41470; Sigma et al., USA) at a dose of 0.3 mg/kg/day diluted in DW to obtain 4 ml/kg via oral gavage for 12 weeks^33^.

*Group VI*: EX and CrM-treated group (EX-CrM): aged rats received CrM and were subjected to combined ladder climbing resistance exercise, as mentioned before, for 12 weeks.

At the end of the experimental period (12 weeks), rats were sedated for electromyography (EMG) recording.

### Measurements of electromyography (EMG)

Electromyographic (EMG) recordings were performed in vivo to detect electrical abnormalities in muscle using the Biopack MP100 acquisition system, Inc., Goleta, California, United States^34^. Two thin concentric needle electrodes were inserted into the gastrocnemius muscle of rats sedated with thiopental sodium (50 mg/kg, i.p., as needed) to measure EMG activity^35^. Two shielded electrodes (LEAD110S/EL503 or EL508S) were used for the signal inputs through a percutaneous puncture in the gastrocnemius muscle, and one unshielded electrode (ground electrode) had been placed in the rat tail. The EMG activity was frequently monitored for approximately three to four minutes following the insertion of the electrodes. Immediately following the test, rats were reintroduced to their enclosures after having fully recovered from anesthesia. A qualitative estimation of myotonic-like activity was derived from integrated EMG parameters involving the mean power, frequency, and duration of electrical activity.

At the end of the experimental period (12 weeks), rats were anesthetized for cardiac puncture with Ketamine (100 mg/ml): Dilute to 50 mg/ml. Draw 1 ml of Ketamine (100 mg/ml) into a sterile syringe and dispense into a sterile multidose vial. To this, add 1 ml of sterile, pyrogen- free water or sterile 0.9% NaCl.

### Collection of blood samples

Via cardiac puncture, 5 ml of blood was taken^36^. Blood from each rat was deposited in a simple, sterile tube and coagulated for roughly 30 min at room temperature. Serum was separated by centrifuging samples at 3000 rpm for 15 min. The samples were kept at − 80 °C for biochemical assessment.

### Biochemical analysis

My BioSource (San Diego, CA, USA) ELISA kits were used to measure the serum levels of TNF-α and IL-6. R&D Systems, Inc. (Minneapolis, MN, USA) ELISA kits were used to measure serum levels of inhibition factor macrophage migration (MIF) and insulin-like growth factor 1 (IGF-1) Abbexa Ltd. (Cambridge, UK) ELISA kits (were used to measure serum levels Of SPARC (osteonectin) and creatine kinase (CK) (Cat.No. abx157210). Serum levels of glutathione reductase (GSH) (Cat.No.GR 25 11) and malondialdehyde (MDA) (Cat.No.MD 25 29) were spectrophotometrically determined using commercial kits supplied by Bio diagnostic, Egypt^37^.

The lipid profile assay was measured by enzymatic colorimetric testing. Serum levels of cholesterol^38^ (Cat. No. CH 12 20), high-density lipoprotein cholesterol (HDL-c)^39^ (Cat. No. CH 12 30), and triglycerides (TGs)^40^ (Cat. No. TR 20 30) were measured by the colorimetric method using the Biodiagnostic kits, Egypt. Low-density lipoprotein cholesterol (LDL-c) was calculated by subtracting total cholesterol (TC), HDL-c, and TG concentrations^41^. An automatic optical reader (SUNRISE Touchscreen, TECHAN, Salzburg, Austria) was used to perform the tests.

### Development of a sarcopenia risk score

Four biomarkers (IL-6, SPARC, MIF, and IGF-1) were used to develop a single risk score^28^. The risk score for each group was calculated as the sum of the risk score for each biomarker, which was derived by multiplying the serum level of a biomarker by its corresponding coefficient (risk score ∑ logistic regression coefficient of biomarker Mi × serum level of biomarker Mi). Rats were diagnosed with sarcopenia using the median cut-off risk score as a threshold^42,43^.

### Quantitative RT-PCR (qRT-PCR)

Each rat had one piece of fresh skeletal muscle tissue collected in a falcon tube and stored at -80 °C for RNA extraction and the assay of NUDT3. NUDT3 was detected using a 7500 real-time PCR system (Applied Biosystems, CA, United States). RNA was extracted from skeletal muscle cells using a direct—zol RNA miniprep kit (Cat. No. R2051; Zymo Research, USA), followed by the first step of PCR: complementary deoxyribonucleic acid (DNA) was synthesized using the QuantiTect Reverse Transcription Kit (205311; Qiagen, Applied Biosystems, USA), and then the second step of PCR (the real-time PCR step): it forward and reverse primers for NUDT3 were 5′-GAGGAGGTACTGCTGGTGAG-3′ and.

5′-TCCCAGCGTCCCTTTTACTC-3′, respectively. As an endogenous control, forward and reverse primers for actin were 5′-GACGGCCAGGTCATCACTAT-3′ and 5′-CTTCTGCATCCTGTCAGCAA -3′.

Each PCR reaction was conducted in a final volume of 20 μL containing 10 μL of SYBR Green (2× Quanti Tect PCR Master Mix), 3 μL of cDNA, 1 μL of forward primer, 1 μL of reverse primer, and 5 ml of RNase-free water. This was followed by 55 cycles of denaturation at 94 °C for 30 s, annealing at 55 °C for 40 s, and extension at 72 °C for 31 s. Version 2.0.1 of the Applied Biosystems 7500 software analyzed the data. Using the comparative Ct method, gene expression was quantified in a relative manner. The amplification plot and melting curve of the NUDT3 gene.

### Histopathological assessment

#### Hematoxylin and eosin staining (H&E)

The selected gastrocnemii muscle tissues were fixed in 10% formaldehyde for 24 h. The samples were embedded in paraffin, cut into four μm-thick sections, and stained using H&E staining according to the routine protocol. The histopathologist assessed slides at different power fields, observing histopathological changes, including fibrosis, inflammation, and adipose tissue formation^44^.

#### Immunohistochemical (IHC) myogenin staining

Four μm-thick sections were cut from paraffin blocks and stained using a fully automated immunohistochemical machine (DAKO) according to the standard IHC protocol. The method used for immunostaining was the streptavidin–biotin amplified system. Antigen retrieval was performed using EDTA buffer at 100 °C for 30 min.

The detection kit is an ultra-vision detection system with anti-polyvalent HRP/DAB (ready to use) (TP-015-HD; Lab Vision, USA). The primary antibody myogenin (a mouse monoclonal antibody; IR067/IS067, DAKO) was used to demonstrate skeletal muscle cell regeneration^45^*.* H&E and immunostained sections were photographed using a Leica inverted microscope.

### Statistical analysis

Statistical analysis was conducted using version 28 of the Statistical Package for the Social Sciences (SPSS) (SPSS Inc., USA). The analysis of the data required descriptive statistics and inferential statistics. Number (No), percent (%), mean (x̅), and standard deviation (SD) were used to express descriptive statistics. Analytic statistics comprised the Kruskal–Wallis (non-parametric), followed by Tamhane's post hoc test for quantitative data and the Chi-square (χ 2) test for examining the relationship between qualitative variables. The odds ratio (OR) was utilized to evaluate the exposure risk. Using regression analysis, predictors of the likelihood of developing sarcopenia (IBS) were identified. Receiver Operator Characteristic (ROC) curve analysis was used to determine the cutoff points, sensitivity, and specificity of the sarcopenia index to detect its utility in predicting muscle mass, and 2 × 2 tables used for the calculation of PPV, NPV, and diagnostic accuracy. A p-value of 0.05 was statistically significant.

### Ethical approval and consent to participate

The ethics committee of the faculty of medicine, Menoufia University Menoufia, Egypt, with approval code 5/2023PHYS16, approved the study.

## Results

### Effects of EX, CoQ10, CrM, and CrM-EX treatment on EMG in studied groups

(Table 1) Electromyography (EMG) (mean, median, peak frequency mv/sec) and mean power and total power (mv) were significantly higher (*P* < 0.05) in the control group when compared to the other groups. EX-CrM group, which in turn showed significant improvement in EMG (*P* < 0.05) compared to the aged, EX, CoQ10-treated, and CrM groups.

**Table 1:** Effect of EX, CoQ10, CrM and EX-CrM on EMG in aged rats.

	CTR	Aged	EX	CoQ10	CrM	EX-CrM
Median frequency (mv/sec)	736.85 ± 4.89	414.97 ± 2.21^a^	88.69 ± 2.70^a^	436.87 ± 20.63^a^	631.27 ± 1.61^abcd^	722.96 ± 3.14^bcde^
Mean frequency (mv/sec)	608.17 ± 2.15	418.66 ± 1.30^a^	425.26 ± 6.88^a^	419.56 ± 4.09^a^	535.17 ± 3.47^abcd^	592.77 ± 2.37^abcde^
Peak frequency (mv/sec)	885.43 ± 2.97	311.13 ± 1.49^a^	502.45 ± 2.36^ab^	393.79 ± 3.13 ^abc^	588.48 ± 2.72 ^abcd^	786.44 ± 3.71 ^abcde^
Mean power (mv)	7.76 ± 0.25	1.26 ± 0.08^a^	1.61 ± 0.32^a^	1.50 ± 0.23^a^	4.08 ± 0.30 ^abcd^	6.48 ± 0.36 ^abcde^
Total power (mv)	5.31 ± 0.22	1.60 ± 0.17^a^	1.76 ± 0.21^a^	1.71 ± 0.18^a^	3.59 ± 0.22 ^abcd^	4.83 ± 0.11 ^abcde^

Data are expressed as mean ± SD (n = 10) and analyzed using Kruskal–Wallis, followed by Tamhane’s post hoc test.

CTR, control; EX, exercise; CoQ10, co-enzyme Q10D; CrM, creatine monohydrate.

^a^(P < 0.05) versus control group; ^b^(*P* < 0.05) versus aged rats group; ^c^(*P* < 0.05) versus EX-treated group; ^d^(^P^*P* < 0.05) versus CrM group; ^e^(*P* < 0.05) versus EX-CrM group.

### EX-CrM combined treatment ameliorates the effect of aging on altered serum lipid profiles in rats

Significant differences (*P* < 0.05) in serum lipid levels (cholesterol, LDL-c, TGS, and HDL-c) were seen in the aged, EX, CoQ10, and CrM groups compared to the CTR group (Table 2).

**Table 2:** The effect of EX, CoQ10, CrM and EX-CrM on lipid profile in aged rats.

	CTR	Aged	EX	CoQ10	CrM	EX-CrM
Cholesterol	60.98 ± 0.81	91.42 ± 1.90^a^	88.69 ± 2.70^a^	87.80 ± 5.99^a^	65.09 ± 0.64^abcd^	60.85 ± 0.57^bcde^
LDL-c	19.96 ± 0.51	32.27 ± 1.15^a^	30.11 ± 1.79^a^	22.72 ± 0.68 ^abc^	21.67 ± 0.86^bc^	19.61 ± 1.27^bcd^
HDL-c	27.75 ± 0.62	16.19 ± 0.25^a^	16.52 ± 0.24^a^	22.45 ± 0.54^abc^	25.30 ± 0.52^abcd^	26.56 ± 0.33^abcde^
TGS	59.31 ± 0.45	72.96 ± 0.55^a^	71.87 ± 1.12^a^	71.57 ± 1.18^a^	60.94 ± 0.81^abcd^	59.83 ± 0.44^bcd^

Data are expressed as mean ± SD (n = 10) and analyzed using Kruskal–Wallis, followed by Tamhane’s post hoc test.

CTR, control; EX, exercise; CoQ10, co-enzyme Q10D; CrM, creatine monohydrate.

^a^(*P* < 0.05) versus control group; ^b^(*P* < 0.05) versus aged rats group; ^c^(*P* < 0.05) versus EX-treated group; ^d^(*P* < 0.05) versus CrM group; ^e^(*P* < 0.05) versus EX-CrM group.

The serum lipid profile of the EX-treated group of the EX-treated group was not significantly different from the control group (*P* > 0.05).

The CrM group serum lipid profile significantly (*P* < 0.05) improves cholesterol, HDL, and LDL levels compared to the aged group.

Similarly, CoQ10 treatment significantly (*P* < 0.05) improves HDL and LDL levels compared to the aged group, but not cholesterol or TGS levels.

EX-CrM improved the lipid profile with no significant difference (*P* > 0.05) from the CTR except for HDL-c.

### CrM-EX combined treatment ameliorates the effect of aging on altered oxidative stress and inflammatory markers (Fig. 1)

**Figure 1:**
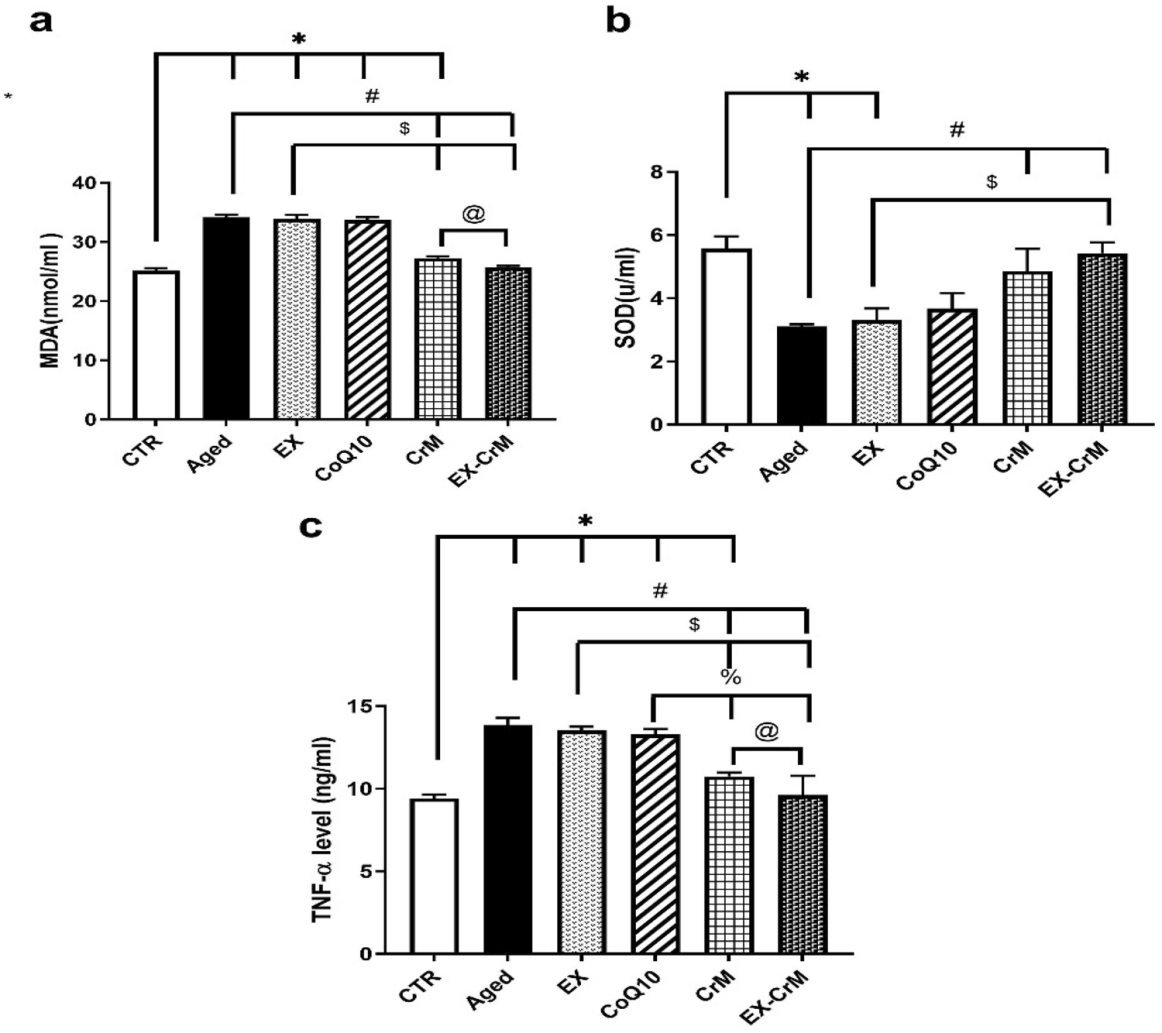
Effect of EX-CrM combined treatment ameliorating effect on aging altered oxidative stress and inflammatory markers (a) Serum malondialdehyde (MDA) level. (b) Serum superoxide dismutase (SOD) level. (c) Serum tumor necrosis factor-α (TNF-α) level. Data were expressed as mean ± SD and analyzed by Kruskal–Wallis, followed by Tamhane’s T2 test (n = 10). *P < 0.05 versus the CTR group; # *P* < 0.05 versus the aged group; $ *P* < 0.05 versus the EX-treated group; % *P* < 0.05 versus the CoQ10-treated group; and @ *P* < 0.05 versus the CrM-treated group.

The serum levels of MDA in the EX-CrM-treated group (25.73 ± 0.24 nmol/ml) were significantly decreased (*P* < 0.05) compared to the corresponding values in the aged, EX, and CoQ10-treated groups. There was no significant change (*P* > 0.05) between the EX-CrM, CrM-treated, and CTR groups.

SOD serum levels in the EX-CrM-treated group (5.43 ± 0.34 u/ml) were significantly increased (*P* < 0.05) compared to the corresponding values in the aged and EX-treated groups. There was no significant change (*P* > 0.05) between the EX-CrM, CrM, CoQ10-treated, and CTR groups.

The serum levels of TNF-α in the EX-CrM-treated group (9.62 ± 1.17 ng/ml) were significantly decreased (*P* < 0.05) compared to the corresponding values in the aged, EX, CoQ10, and CrM-treated groups. There was no significant difference (*P* > 0.05) between the EX-CrM-treated and CTR groups.

### Effects of EX, CoQ10, CrM, and EX-CrM treatment on serum CK levels and on the NUDT3 gene in studied groups

**Figure 2:**
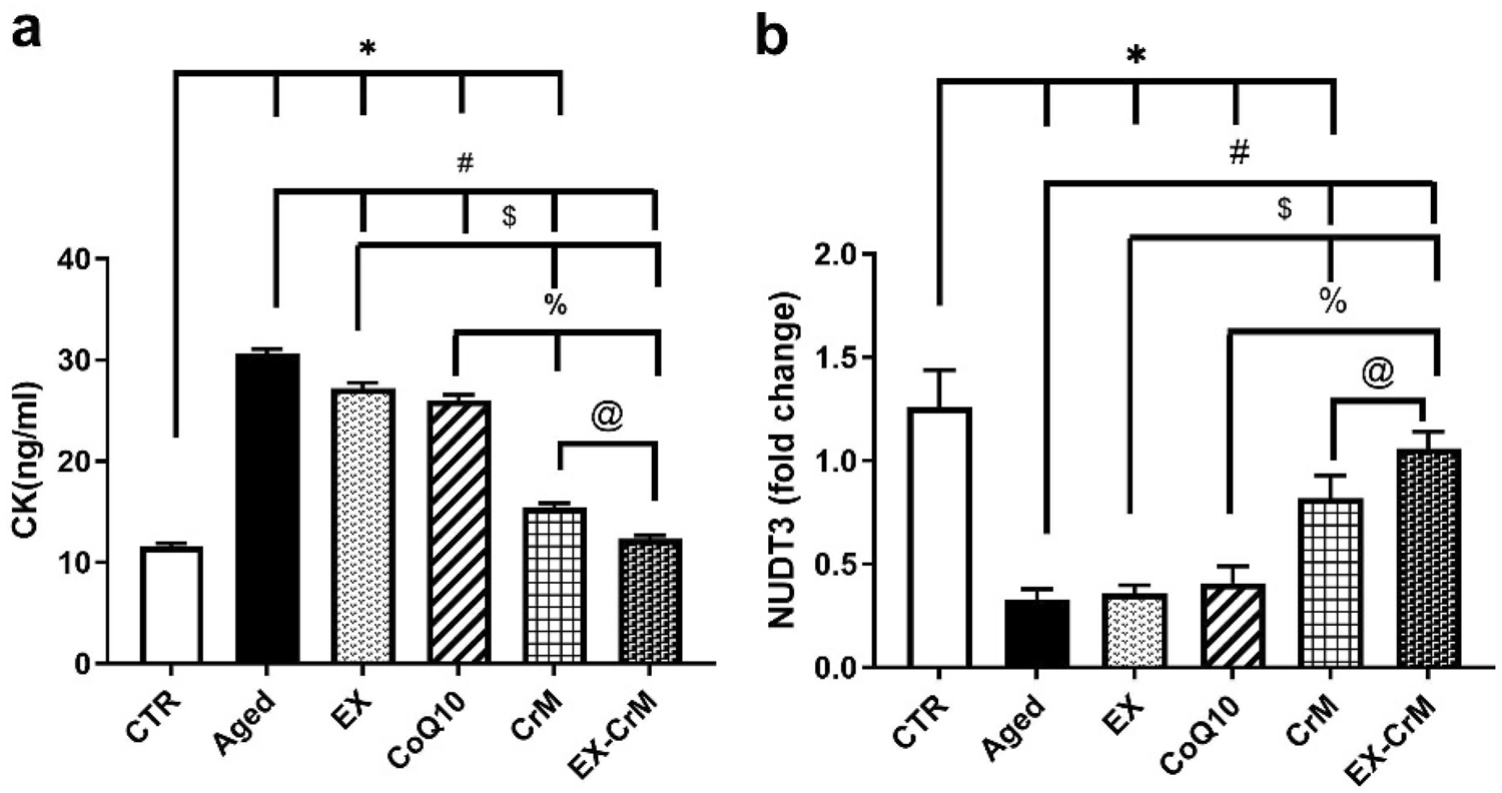
Effects of EX, CoQ10, CrM, and EX-CrM treatment on serum CK levels and on the NUDT3 gene in studied groups. (**a**) Serum creatine kinase (CK) level. (**b**) NUDT3 expression in skeletal muscle. Data were expressed as mean ± SD and analyzed by Kruskal–Wallis, followed by Tamhane’s T2 test (n = 10). *P < 0.05 versus the CTR group; # *P* < 0.05 versus the aged group; $ *P* < 0.05 versus the EX-treated group.

Figure 2 showed there was a significant difference (*P* < 0.05) in serum CK (ng/ml) in the EX, CoQ10, CrM, and EX-CrM treated groups (27.21 ± 0.54, 26.54 ± 0.42, 15.39 ± 0.45, and 12.31 ± 0.37 ng/ml) when compared to the corresponding values in the aged group (30.62 ± 0.47). There was a nonsignificant difference in the CK level of the CoQ10 group compared to the EX-treated group (*P* > 0.05). There was a significant increase in CK levels in the EX, CoQ10, CrM, and EX-CrM groups compared to the CTR group (*P* < 0.05).

The group of aged rats showed a significant reduction in skeletal muscle NUDT3 expression compared to the control group (*P* < 0.05). CrM- and EX-CrM-treated groups showed a significant (*P* < 0.05) increase in NUDT3 expression compared to the aged rats group. EX- and CoQ10-treated groups showed a nonsignificant increase in NUDT3 expression compared to the aged group (*P* = 0.524 and *P* = 0.232, respectively).

### Effects of EX, CoQ10, CrM, and CrM-EX treatment on sarcopenic index markers in studied groups

**Figure 3:**
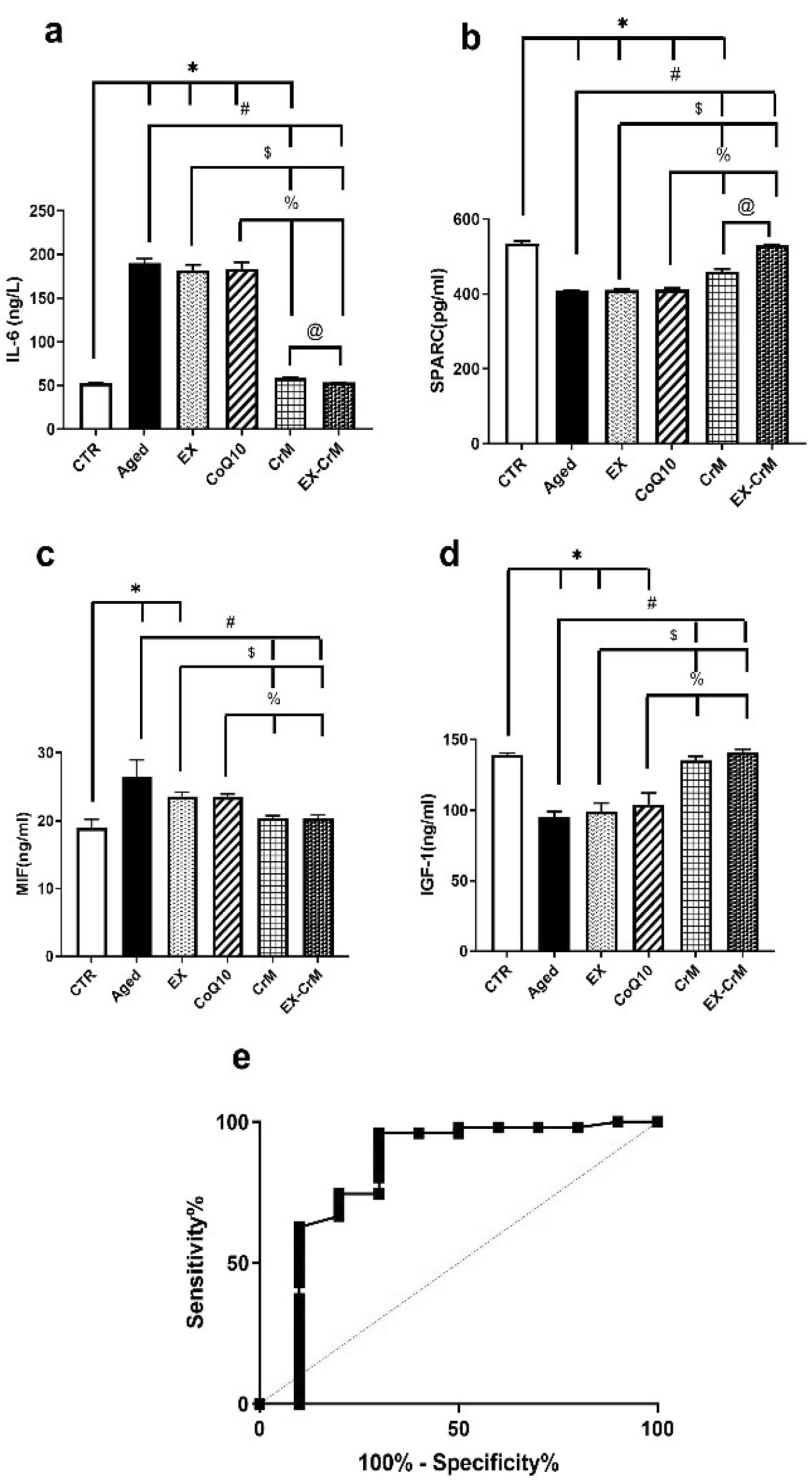
The effect of EX, CoQ10, CrM, and EX-CrM on sarcopenia risk score parameters in aged rats. (**a**) Serum interleukin 6 (IL-6) level. (**b**) Serum secreted protein acidic rich in cysteine (SPARC) level. (**c**) Serum macrophage migration inhibitory factor (MIF) level. (**d**) serum insulin-like growth factor-1 (IGF-1) level. (**e**) Receiver-operating characteristic (ROC) curve of the sarcopenia risk score. Data were expressed as mean ± SD and analyzed by Kruskal–Wallis, followed by Tamhane’s T2 test (n = 10). *P < 0.05 versus the CTR group; # *P* < 0.05 versus the aged group; $ *P* < 0.05 versus the EX-treated group; % *P* < 0.05 versus the CoQ10-treated group; and @ *P* < 0.05 versus the CrM-treated group.

Table 3 showed a nonsignificant difference in IL6, IGF1, SPARC, and MIF serum markers for sarcopenia in the EX- and CoQ10-treated groups compared to corresponding values in the aged group (*P* > 0.05). There was a significant change (*P* < 0.05) in sarcopenia index markers in the combined EX-CrM-treated group compared to the aged, EX, and coq10-treated groups. There was a nonsignificant change in sarcopenia index serum markers in the EX-CrM-treated group (*P* > 0.05) compared to the CTR group. MIF serum level was non-significantly decreased compared to the CrM-treated group (Fig. 3a–d).

**Table 3:** Univariate regression analyses of 4 proteins.

Protein	Estimated β	Std. error	*P* value
IL-6	0.195	0.081	0.019
SPARC	0.146	0.078	0.045
MIF	0.315	0.131	0.002
IGF-1	-0.128	0.116	0.006

*β indicates regression coefficient value.

### Diagnostic validity of sarcopenia index

Receiver Operator Characteristic (ROC) curve analysis was used to detect the utility of the sarcopenia index to predict muscle mass. The Sarcopenia index showed a diagnostic accuracy of 78% at a 95% confidence interval, 79% sensitivity, 71% specificity, 76% Positive predictive value (PPV), and 80% negative predictive value (NPV) at the cut-off point of 2.369 (Fig. 3e).

### Histopathological examination

#### H&E staining

**Figure 4:**
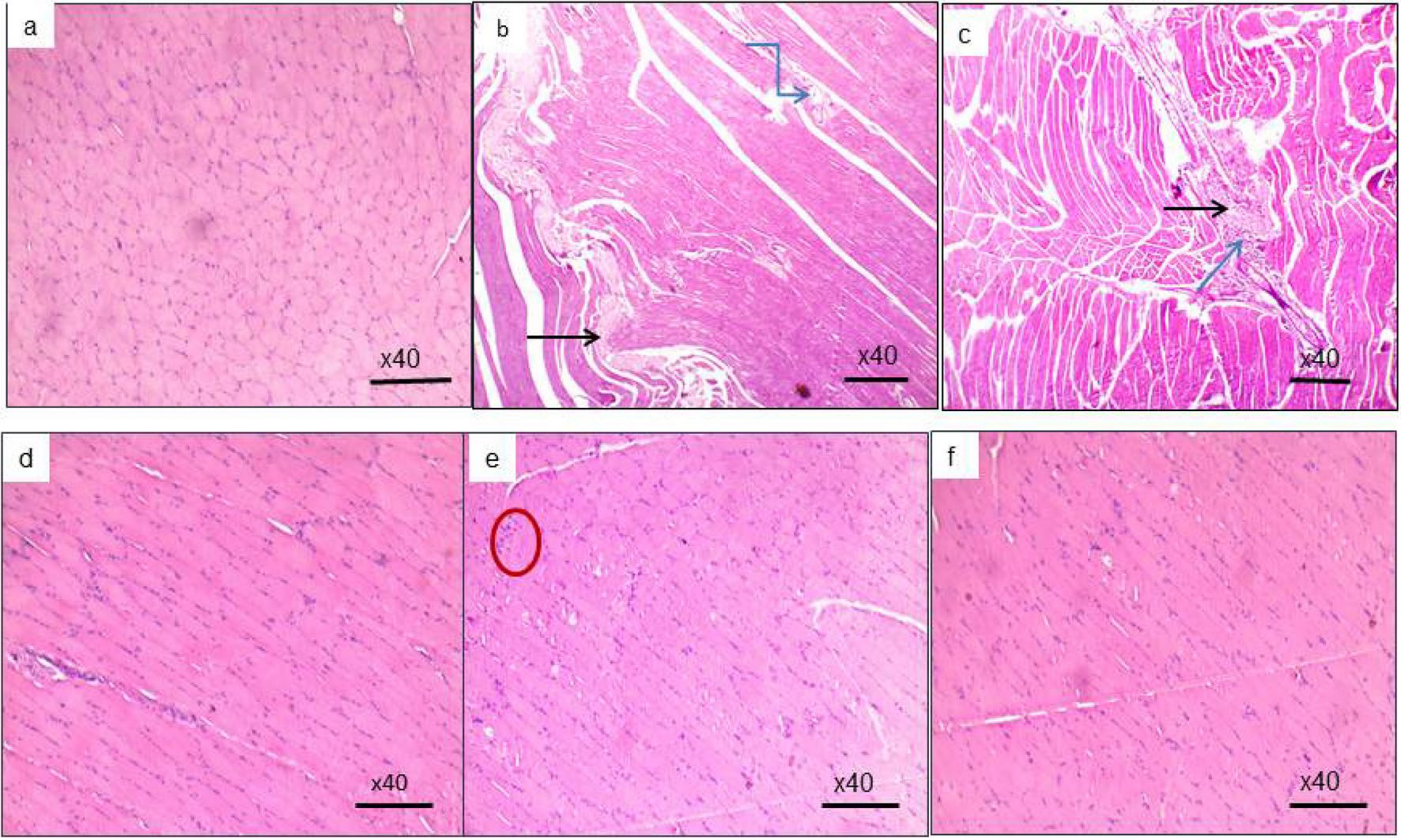
H&E-stained sections (**a**) The CTR group exhibited a normal histological microscopic picture of skeletal muscle in syncytia with multiple eccentric nuclei and cross striations (original magnification ×40). (**b**) Fibrous tissue (black arrow) extending broadly between skeletal muscle fibers showed a decreased number of nuclei (muscle mass) with adipose tissue (blue arrow) in the aged group (original magnification ×40). (**c**) The EX-treated group showed fibrous tissue (black arrow) together with chronic inflammation (blue arrow) and chronic inflammation in between muscle fibers (original magnification ×40). (**d**) The CoQ10-treated group showed slight chronic inflammation and larger muscle mass (increased nuclei number) than the aged group (original magnification ×40). (**e**) The CrM-treated group started to have suspected regeneration by the appearance of multiple myoblast-looking cells (cells with single round nuclei, red circles) (original magnification ×40). (**f**) The EX-CrM-treated group featured a histologic appearance almost like the control group's skeletal muscle fibers (original magnification ×40).

On the evaluation of H&E-stained sections, the aged group showed pathological changes in chronic inflammation, fibrous tissue, and fat deposited in between muscle fibers, together with a decreased number of peripheral nuclei of muscle fibers. Sections examined from the three treated groups, mostly aged combined EX and CrM treated groups, exhibited minimal to the absence of the previously mentioned pathological changes with the initiation of muscular regeneration and returning to the average adult skeletal muscle histological appearance (Fig. 4).

#### Myogenin expression

**Figure 5:**
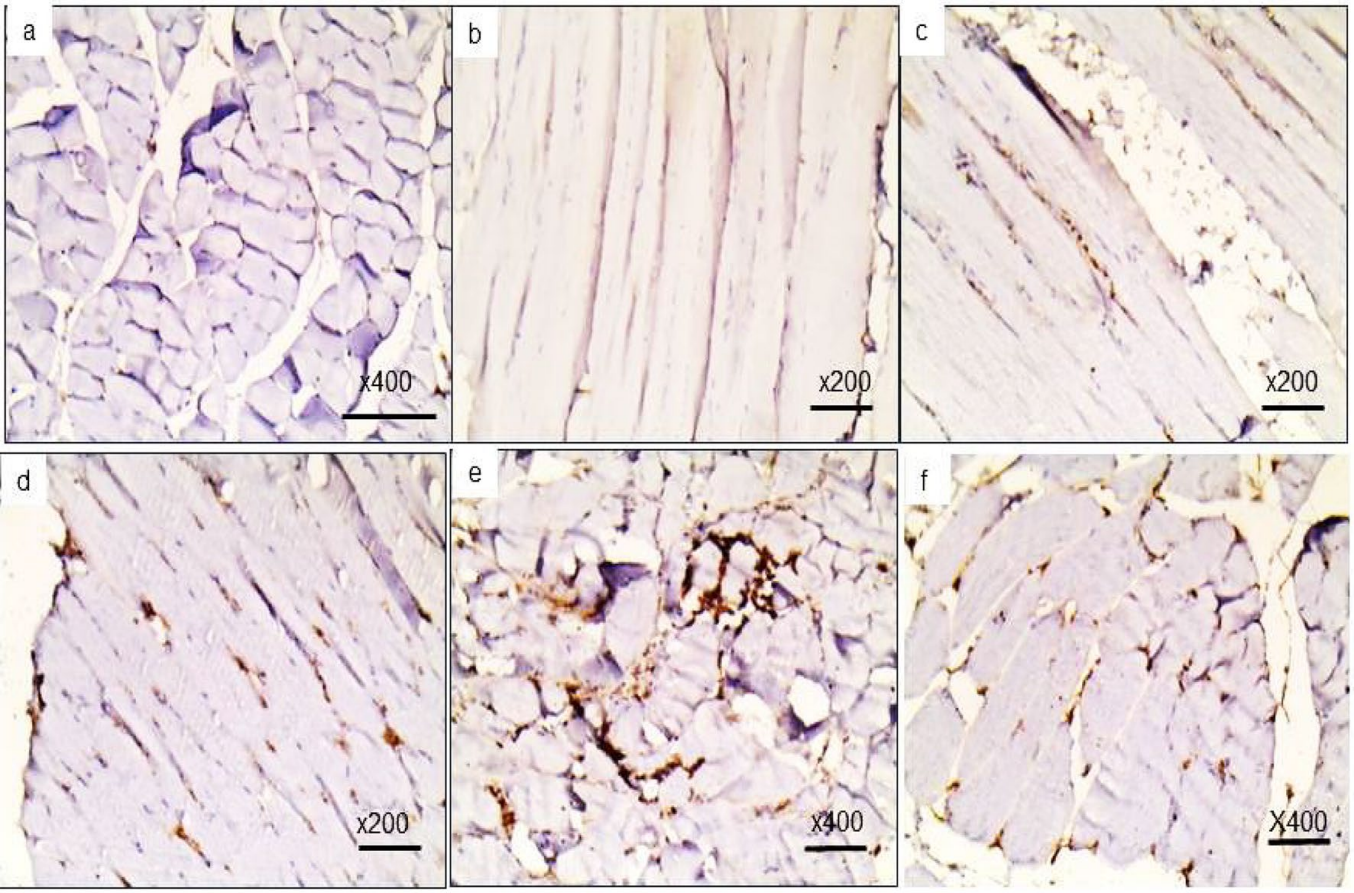
Myogenin immunostaining. (**a**) Focal myogenin nuclear expression (×400) in the CTR group. (**b**) Negative myogenin expression in the aged group (×200). (**c**) Mild nuclear myogenin expression in the EX-treated group (×200). (**d**) Myogenin nuclear expression demonstrated stimulated early muscle differentiation (myoblasts) in the CoQ10-treated group (×200). (**e**) The crM-treated group showed moderate to strong myogenin immunoexpression (×400). (**f**) Highest Myogenin nuclear expression in the EX-CrM-treated group (×400).

Myogenin expression in the aged group was almost negative compared to the CTR group. Treated groups showed higher levels of myogenin expression than the aged group, with the highest in the EX-CrM-treated group. (Fig. 5).

## Discussion

Age-related sarcopenia is considered a disability. Consequently, increasing socioeconomic burdens on the healthcare system are detrimental to society. As a result, a growing array of studies on sarcopenia, adopting animal models to investigate novel therapeutic approaches to delay its impact^46^, are being conducted.

Metabolic disorders such as dyslipidemia, hypertension, and sarcopenia have been linked to aging^47^. According to this study, aged rats had considerably higher mean blood cholesterol, TGs, and LDL-c values than the CTR group. Consistent with our results*, *Liu and Li^48^ suggested that the primary cause of aging hypercholesterolemia is a steady decline in the liver's ability to convert plasma cholesterol into bile acid. Regarding the increased levels of LDL-c and TG in the aged rats, Jia et al.^49^, Perdomo and Henry Dong^50^ revealed that a loss in both oxidative capacity and mass of metabolically active tissues with aging was also connected with an increase in the release of free fatty acids (FFAs) from adipocytes, which contributed to hyperinsulinemia, insulin resistance, and hypertriglyceridemia.

In the current study, the HDL-c level was significantly decreased in the aged rats compared to the CTR group, and this finding was supported by Walter et al.^51^, who stated that the aging process is correlated with hormonal changes, inflammatory processes, and insulin resistance that impair lipolysis. Additionally, aging may affect reversed cholesterol transport (RCT), a critical factor in determining HDL-c levels^52^. Inflammatory processes in the aged population and declining testosterone levels may cause low HDL-c by physically replacing apolipoprotein A-I with the acute-phase reactant serum amyloid A and various cytokine-induced changes^53,54^.

Our data substantiate the beneficial effects of combined EX and CrM treatment, which showed a significant improvement in the lipid profile compared to the corresponding values of the aged group. This change may be due to Cr supplementation enhancing high-intensity exercise performance^22^ (Supplementary Fig. S1).

The improvement in the CrM-treated group's lipid profile was significant compared to the aged group. This finding, agreed with Walsh et al.^55^, revealed that improvement may be due to its antioxidant effect as well as the fact that the fact that CrM stimulates mitochondrial oxidative phosphorylation and could indirectly affect the plasma lipid profile.

The current study concluded that the CoQ10 group had an improvement in HDL-c and LDL-c, but not in all aspects of their lipid profiles. Unfortunately, Liu et al.^56^ stated that CoQ10 improves the lipid profile as it enhances cellular antioxidant status and reduces the concentration of hepatic conjugated dienes through its action on peroxisome proliferator-activated receptors (PPAR), inhibiting lipid peroxidation^57^. These results were not aligned with our results, which may be due to using a larger sample or a longer duration.

Sarcopenia raises creatinine and CK. Sarcopenia can be distinguished from other muscular degenerative illnesses using it^58^. We found that aged rats had higher CK serum levels than CTR animals. Prajapati et al.^59^ reported similar results: CK rose due to muscle fiber loss. CK levels in the EX-CrM-treated group were lower than those in the aged rat group, but CoQ10-treatment did not matter.

In terms of inflammation, the aged group had significantly higher levels of serum IL-6 and TNF-α compared to the CTR group. Aging may increase total, visceral, and truncal fat. Central adiposity produces cytokines and adipokines, such as IL-6 and TNF-α^60^. Increased levels of serum IL-6 can cause decreased muscle mass and muscle power, along with decreased physical performance^61,62^. A control policy of aging with exercise may ameliorate the inflammatory state, as found in this study, but it is still significantly higher than the control group. Exercise contributes to the anti-inflammatory effects associated with aging^63^.

A nonsignificant elevation in serum inflammatory markers was observed in the CoQ10-treated group compared to the aged rats' group. CoQ10 supplementation improves mitochondrial function, which reduces the chronic oxidative stress linked to many degenerative diseases and enhances aged individuals' health^64,65^.

In the current study, treatment with Cr or combination therapy with exercise showed more improvement in the inflammatory state. Indeed, there is much evidence showing that modulating lifestyle through exercise improves life quality as it mediates muscle health and longevity^66^, particularly resistance exercise^67^.

Exercise affects muscle function by modifying exercise-responsive gene expression (i.e., PGC-1α, TFAM, and MEF2A), even after exercise, triggering structural and metabolic adaptations in skeletal muscles^68^. Also, it mediated muscle health and longevity through sirtuin-1-regulated pathways and the regulation of PGC-1α, the master controller of mitochondrial biogenesis^30^. Mitochondrial morphological changes decrease inflammatory and oxidative states in old age due to increasing nuclear respiratory factor (NRF-1) levels. This results in increased mitochondrial oxidative capacity and ATP production^69^.

Our results indicate that serum biomarkers correlate with the metabolism and function of skeletal muscle. IL-6 was the first myokine to be identified^70^. Serum IL-6 has been identified as a potential biomarker for sarcopenia^71^. Puzianowska-Kunicka et al.^72^ found substantially higher IL-6 levels in older individuals due to the overproduction of oxygen-free radicals^71,73^, corroborating our findings.

In our study, the serum levels of SPARC and IGF1 in aged rodents were significantly lower than in the CTR group. The SPARC level increased, but not significantly, when comparing the EX-treated group to the aged group. This change may be the result of a slight increase in SPARC expression in activated (exercised) skeletal muscle. The 5' AMP-activated protein kinase (AMPK) is a potent signal related to exercise intensity. The activity of this enzyme increases the expression of PGC-1^68,74^ based on the intensity of the exercise.

IGF1, also referred to as somatomedin C, is a well-known muscle growth and regeneration^75^ mediator. IGF-1 signaling involves the activation of phosphatidylinositol-3-kinase (PI3K), resulting in muscle hypertrophy via stimulation of protein synthesis pathways and prevention of muscle atrophy pathways, and causing human myotube hypertrophy by accelerating the recruitment of reserve cells^76,77^. This was in line with the findings, which demonstrated a considerable decrease in IGF1 in the aged group compared to the CTR group. Moreover, IGF-1 is present in human skeletal muscle.

MIF is a pro-inflammatory cytokine that affects muscle injury and glucose homeostasis. This result was in line with the findings of the current study. Compared to the CTR group, MIF dramatically increased in the aged rats. The skeletal muscle is a crucial organ for using glucose. We hypothesize that the metabolic status in sarcopenia may be reflected in circulating MIF levels^78^.

In the present study, the CrM-treated group significantly increased SPARC levels compared to the corresponding values of the CoQ10-treated group. CrM, concurrently with resistance exercise, amplifies the physiological reduction in myostatin that decreases skeletal muscle growth with resistance exercise alone^79^. CrM produces a minimal increase in strength in patients with muscular dystrophy^80^.

Recently, skeletal muscle has gained much attention as an endocrine organ that secretes myokines. Exercise significantly decreased IL-6 compared to the aged rats' group, as the oxidative level increased with exercise due to the activation of nuclear factor-κB, which mediates the secretion of IL-6. Muscle contraction releases calcium ions, promoting IL-6 secretion^81^. Moreover, Gilmartin et al.^82^ confirmed that exercise could induce MIF and SPARC protein secretion from skeletal muscle.

In this study, there was a significant decrease in the mean values of the mean power and mean frequency of EMG in the aged group compared to the CTR group. This finding is supported by Plate et al.^83^, who revealed that aging decreases the amounts of myogenic regulatory factors (Myogenin, MyoD, and Myf5) and reduces functional and regenerative capacity. The myogenin gene is indispensable for skeletal muscle development^84^. The expression of myogenin is highly restricted to myogenic tissues in embryonic, fetal, and adult skeletal muscles. Myogenin is absent in undifferentiated cells, peaks, and then declines following a stimulus to differentiate, and is overexpressed in myoblasts^85^.

Combined EX-CrM treatment significantly increased the mean power and mean frequency of muscle contraction of EMG when compared to the aged group, explained by the anti-inflammatory effects of CrM in addition to physical exercise-mediated improvement in muscle health and longevity^86^.

Multifactors contribute to mitochondrial dysfunction and injury, including ROS-induced damage caused by aging and a decline in antioxidant enzyme levels. Fortunately, this was ameliorated by exercise and CrM supplementation^69^. CrM supplementation influences muscle mass and performance positively^87^. CrM has been shown to promote myogenesis by increasing the level of expression of specific muscle regulatory factors that exert trophic, antioxidant, and anti-inflammatory effects on muscle cells^88^.

The study of Jin et al.^12^ represented a regulatory function for the association of NUDT3 with sarcopenia and found that its expression was related to LBM. They identified NUDT3 as a significant biomarker for LBM, and in the study of Singh and Gasman^11^, they found that NUDT3 is related to actin production in the muscle tissue.

Research has yet to be done on how treatment with Cr or coQ10 affects NUDT3's gene expression. CoQ10 may help with effective aging control or prevent age-related change^89^.

Following our findings, EX and CrM supplementation significantly ameliorate NUDT3 compared to the aged group. This finding may be due to increased intramuscular water, inhibition of protein breakdown and RNA degradation, and stimulation of protein, DNA, RNA, and glycogen synthesis^90^.

This suggests a new physiological role for CrM, pointing to its many impacts beyond ergogenicity. Nutritional supplements like CrM may postpone the start and course of sarcopenia^91^.

The dietary supplement CrM boosts workout performance. Researchers propose oral CrM supplementation may boost skeletal, cardiac, and smooth muscle creatine^92,93^. CrM supplementation may increase muscular ATP in high-intensity, short-term exercise demands that rely on phosphocreatine^94,95^. CrM has antioxidant properties because it induces peroxiredoxin-4 and thioredoxin-dependent peroxide reductases and activates AMPK, which promotes adaptive cellular responses to oxidative stress. Supplemental CrM may boost intracellular arginine, an antioxidant^69^.

## Conclusion

In the present study, NUDT3's and myogenin have demonstrated efficacy as diagnostic methods for sarcopenia. The EX-CrM combination showed a notable improvement in the lipid profile and antioxidant and anti-inflammatory effects. Also, this combination improved muscle power and frequency (EMG) compared to the EX-, CoQ10-, and CrM-treated groups. Therefore, the EX-CrM combination could be recommended for treating and controlling age-related sarcopenia. These results may be due to the additive effect of CrM on EX treatment in improving muscle ATP content, vascularity, and reduction of ROS. Despite the limitations of the current study of sample size, more groups are needed in the study design. Our results clearly support a new innovative approach to molecular diagnosis of sarcopenia, providing a step in further understanding the mechanisms underlying sarcopenia and identifying novel therapeutic lines.

## Data availability

The datasets used and analyzed during the current study are available from the corresponding author upon reasonable request.

### Supplementary Information

Supplementary Figure S1.
