# Supplementary material for: Comparing the efficacy of concomitant treatment of resistance exercise and creatine monohydrate versus multiple individual therapies in age related sarcopenia
Source: Sci Rep. 2024 Apr 29;14:9798. doi: 10.1038/s41598-024-59884-w (PMC11058861; doi:10.1038/s41598-024-59884-w)
Supplement: Supplementary file 1 — Supplementary Figure S1. [file 41598_2024_59884_MOESM1_ESM.docx]

**
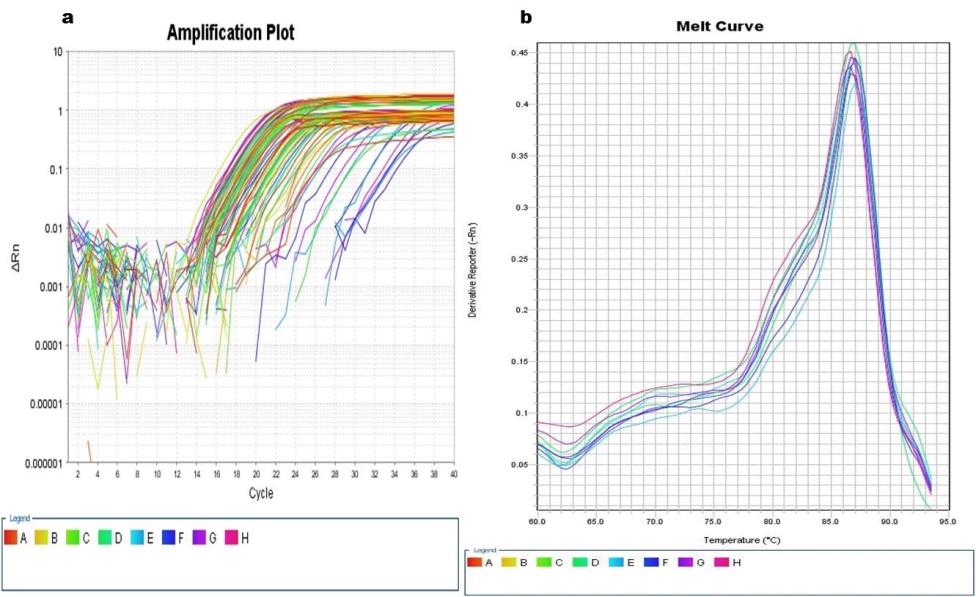
**

**Figure:**

Effect of EX-CrM combined treatment ameliorating effect on aging altered NUDT3 expression (a) Amplification plot of NUDT3 gene expression.

(b) Melting curve of NUDT3 gene expression.

Bars from A-H resemble the rows of real time PCR plate
